# Incorporation of Fluoride into Human Teeth after Immersion in Fluoride-Containing Solutions

**DOI:** 10.3390/dj10080153

**Published:** 2022-08-17

**Authors:** Jana Storsberg, Kateryna Loza, Matthias Epple

**Affiliations:** Inorganic Chemistry and Center for Nanointegration Duisburg-Essen (CENIDE), University of Duisburg-Essen, Universitaetsstrasse 5-7, 45117 Essen, Germany

**Keywords:** fluoride, electron microscopy, enamel, dental care, X-ray powder diffraction, tooth mineral

## Abstract

Toothpastes and mouth rinses contain fluoride as a protective agent against caries. The aim of this study was to determine the degree of fluoride-uptake by human tooth mineral during immersion into fluoride-containing aqueous solutions as different pH. Human teeth were immersed in fluoride-containing solutions to assess the extent of fluoride incorporation into tooth enamel. A total of 16 extracted teeth from 11 patients were immersed at 37 °C for one minute into aqueous fluoride solutions (potassium fluoride; KF) containing either 250 ppm or 18,998 ppm fluoride (1-molar). Fluoride was dissolved either in pure water (neutral pH) or in a citrate buffer (pH 4.6 to 4.7). The elemental surface composition of each tooth was studied by energy-dispersive X-ray spectroscopy in combination with scanning electron microscopy and X-ray powder diffraction. The as-received teeth contained 0.17 ± 0.16 wt% fluoride on average. There was no significant increase in the fluoride content after immersion in 250 ppm fluoride solution at neutral or acidic pH values. In contrast, a treatment with a 1-molar fluoride solution led to significantly increased fluoride concentrations by 0.68 wt% in water and 9.06 wt% at pH 4.7. Although such fluoride concentrations are far above those used in mouth rinses or toothpastes, this indicates that fluoride can indeed enter the tooth surface, especially at a low pH where a dynamic dissolution-reprecipitation process may occur. However, precipitations of calcium fluoride (globuli) were detected in no cases.

## 1. Introduction

Caries still has a high prevalence worldwide, affecting both adults and children [1,2,3]. To prevent this disease, several strategies have been developed. An important factor to reduce the risk of caries is diet with a low sugar content [4]. Additionally, different preventive measures can be applied for home and professional care. They can be broadly divided into plaque removal and topical application of remineralizing agents [1]. To control dental plaque, antibacterial compounds such as chlorhexidine (CHX; 0.2%) and cetylpyridinium chloride can be used [5,6,7,8,9]. Mechanical plaque control can be achieved with tooth brushing, professional tooth cleaning, and flossing [10,11,12].

The inorganic mineral in human teeth is hydroxyapatite, Ca_5_(PO_4_)_3_OH, with small ionic substitutions (“bioapatite”) [13]. As acid-soluble mineral, it is subjected to erosion by the attack of acidic agents, including caries [14]. To increase tooth remineralization and to minimize demineralization, products based on fluorides and/or calcium phosphates are used [3,15,16,17,18,19]. Of all remineralization agents, fluoride is most commonly applied [1,10]. It is used in different products, e.g., in mouthwashes [20], toothpastes [15], gels [21], and varnishes [22] in varying concentrations for caries prevention [23]. Fluoride-free prophylactic systems for dental care involve mainly calcium phosphate-based formulations where calcium phosphate is applied to improve and restore the quality of the inorganic tooth mineral [16,17].

Oral care products can contain different fluoride sources, for example, sodium fluoride (NaF), sodium monofluorophosphate (Na_2_PFO_3_), tin(+II)fluoride (SnF_2_), and amine fluorides (R_4_N^+^F^−^) [10,24]. In Germany, fluoride mouthwashes for adults contain approx. 220–500 ppm fluoride, and fluoride toothpastes for adults contain usually 1450 ppm fluoride (both classified as cosmetic products for daily application). Products with higher fluoride concentrations (classified as drugs) are used in special cases. For example, fluoride gels contain 12,500 ppm F^−^ (application once per week), and fluoride varnishes (application approx. once or twice per year) contain even higher fluoride concentrations (depending on the manufacturer, e.g., 22,600 ppm F^−^) [25,26]. For toothpastes, it is important to note that calcium-containing abrasives such as calcium carbonate may reduce the availability of fluoride due to precipitation of calcium fluoride (CaF_2_) [27,28]. Different modes of action of fluorides on teeth have been postulated, for example, the formation of protecting layers of fluoroapatite, Ca_5_(PO_4_)_3_F, and of calcium fluoride, CaF_2_ [1,29,30]. However, the fluoridation is limited to the outermost tooth surface [31] (see [14] for a recent review on the state of evidence about the physico-chemical fluoride incorporation into the tooth surface). In general, it was put into question whether a very thin fluoride-rich layer can protect teeth from the attack of acids [32].

The aim of this study was to analyze the effect of fluoride treatment on the human enamel surface by energy-dispersive X-ray spectroscopy (EDX), scanning electron microscopy (SEM), and X-ray powder diffraction (XRD). In particular, the incorporation of fluoride was assessed.

## 2. Materials and Methods

A total of 16 extracted teeth from 11 adult patients were obtained from private dentistry practices after medically caused extraction. The teeth were cleaned and stored in water after extraction. No patient information was recorded except for the fact that all patients were adults with a regular history of toothbrushing/dental care. The teeth were selected according to their preservation state. The teeth were mostly caries-free and had no tooth fillings. The teeth were rinsed with water before the subsequent immersion experiments. The samples were randomly divided into 5 groups with 3 teeth for each the immersion experiments. The teeth were placed in the solution at 37 °C for one minute and then thoroughly rinsed with distilled water and air-dried.

The following aqueous immersion solutions were used to simulate treatment with fluoride-containing oral care products:

- 250 ppm potassium fluoride (KF) in water (pH = 5.85)
- 250 ppm potassium fluoride (KF) in citrate buffer (pH = 4.6)
- 18,998 ppm potassium fluoride (1 mol L^−1^ KF) in water (pH = 7.76)
- 18,998 ppm potassium fluoride (1 mol L^−1^ KF) in citrate buffer (pH = 4.7)
- fluoride-free citrate buffer (pH = 4.6)
- water (pH ca. 7; one tooth as control)

A pH of 4.6 to 4.7 was adjusted with a citrate buffer (citric acid 54.1 mM/sodium citrate 46 mM). Potassium fluoride (Acros Organics—Fisher Scientific, 99% extra pure, Schwerte, Germany), sodium citrate dihydrate (AppliChem, 99%, Darmstadt, Germany), citric acid (Honeywell-Fluka, 99.5%, Schwerte, Germany) were used. As solvent, distilled water was used in all cases.

The tooth surface morphology was examined before and after treatment for each tooth by scanning electron microscopy (SEM) with an Apreo S LoVac instrument (Thermo Fisher Scientific, Waltham, MA, USA). The teeth were not sputtered with a conducting metal (as common in SEM) to allow the assessment of the effect of the immersion without artifacts. Instead, they were electrically contacted and grounded to avoid electric charge accumulation. To analyze the chemical composition of the tooth surface including the fluoride content, energy-dispersive X-ray spectroscopy (EDX) was used (UltraDry EDS detector, Thermo Fisher Scientific). For this, 8 to 10 randomly selected spots of the outer enamel surface were analyzed. After fluoride treatment, the surface of the same tooth was again examined at 8 to 10 randomly selected spots by SEM for morphological and chemical changes. For X-ray powder diffraction (XRD), the outer layer of the enamel was ground to powder with a diamond-coated drill to a depth of 0.5 to 1 mm. The obtained powder was analyzed with a Bruker D8 Advance powder diffractometer (Cu Kα radiation, *λ* = 1.54 Å) in Bragg-Brentano reflection mode. For qualitative analysis the Bruker (Billerica, MA, USA) software Diffrac.suite Eva (V6) V1 and the diffraction pattern of synthetic hydroxyapatite (ICDD number 000-09-0432) as reference were used.

For statistical analyses, SPSS (IBM for Windows, version 26, Armonk, NY, USA) was used. The effects of the immersions were investigated separately for each tooth. Exact non-parametric Mann–Whitney tests were performed as the Wilk-Shapiro test indicated a violation of normal distribution in some teeth. Two-sided *p*-values are reported. *t*-tests for independent samples were performed. All errors and error bars are given as standard deviations.

## 3. Results

The human teeth were immersed at 37 °C for 1 min in the different solutions to simulate dental care treatment. Specifically, the solutions with 250 ppm fluoride served as model for mouth rinses, while a highly concentrated potassium fluoride solution (18,998 ppm; 1 mol L^−1^) represented fluoride varnishes. Fluoride was applied in water (approximately neutral pH, slightly changed by the presence of atmospheric carbon dioxide and the dissolved potassium fluoride) and in citrate buffer (pH 4.6 to 4.7). The acidic medium was chosen to mimic the acidic nature of some fluoride gels for which a higher fluoride uptake has been reported [14,26]. To keep the system as simple as possible, fluoride was applied in ionic form as potassium fluoride. Potassium fluoride is a salt that fully dissociates in water, i.e., the molar fluoride concentration is the same as the molar salt concentration. It has the same chemical effect as the salt sodium fluoride (NaF) which is routinely used in dental care, also as an additive to toothpastes. The use of a well soluble salt avoids the chemical dissociation of sodium monofluorophosphate which may cause differences between sodium fluoride and sodium monofluorophosphate. It has been reported that the type of fluoride may also have an influence on the caries-preventing effect, i.e., sodium monofluorophosphate seemed to be less effective compared to sodium fluoride [33]. Taken together, the immersed teeth were subjected to fluoride ions and the stated concentration.

**Figure 1:**
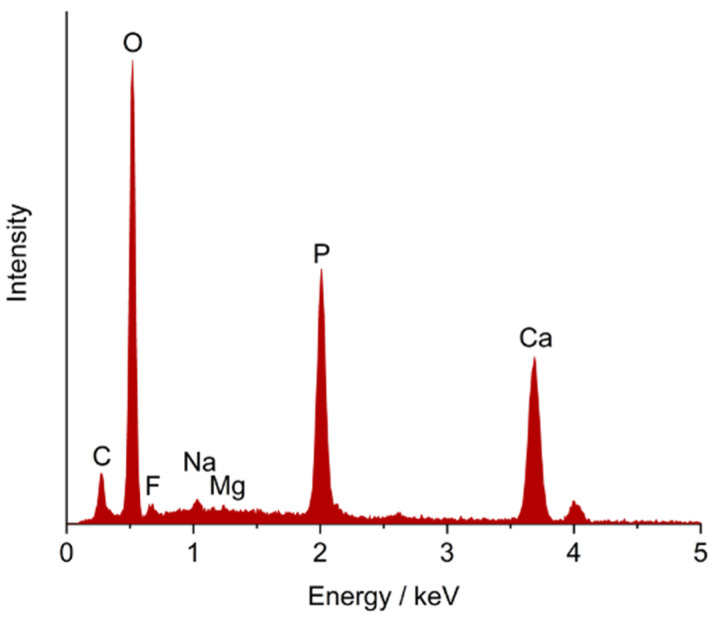
Representative EDX analysis of the enamel surface after treatment with a fluoride solution of 1 mol L^−1^ (18,998 ppm fluoride). Hydroxyapatite in enamel gives rise to signals of oxygen (O), phosphorus (P), and calcium (Ca). Sodium (Na) and magnesium (Mg) are cationic substitutions in hydroxyapatite. Carbon (C) is due to carbonate substitution in hydroxyapatite and the organic part of enamel (matrix proteins). Fluorine (F) is present as an anionic substitution in hydroxyapatite.

The tooth surface was analyzed before and after immersion. Figure 1 shows the elemental composition of a tooth after immersion. As expected for enamel with its high content of calcium phosphate in the form of hydroxyapatite [13], the elements calcium, phosphorus and oxygen were most prominent. The carbon signal is due to the organic part of enamel (matrix proteins) and to substituting carbonate ions in the apatite lattice [13]. Sodium and magnesium are cationic substitutions in hydroxyapatite [13]. Note that EDX is sensitive for the tooth surface with a penetration depth of about 0.1–2 µm (depending on the electron energy and the density of the sample), i.e., we are analyzing only the fluoride content of the outermost surface part of enamel.

Of particular interest in our study is the fluoride content, which is reported with about 0.01 wt% in native teeth [13]. EDX analysis of the as-received teeth showed a low fluoride content despite the year-long use of toothpastes and/or mouth rinses. The mean fluoride concentration was 0.17 wt% with a high standard deviation of 0.16 wt%. This was obviously increased by treatment with fluoride-containing oral care products while the teeth were still functional in the patients′ mouth. As the samples were extracted patient teeth and not standardized enamel samples, this high variation was expected due to different oral care habits of the patients [26,34,35,36]. The reduce the variation between the samples, the fluoride concentration was measured for each individual tooth before and after immersion, and the change in fluoride concentration was considered separately for each tooth.

**Figure 2:**
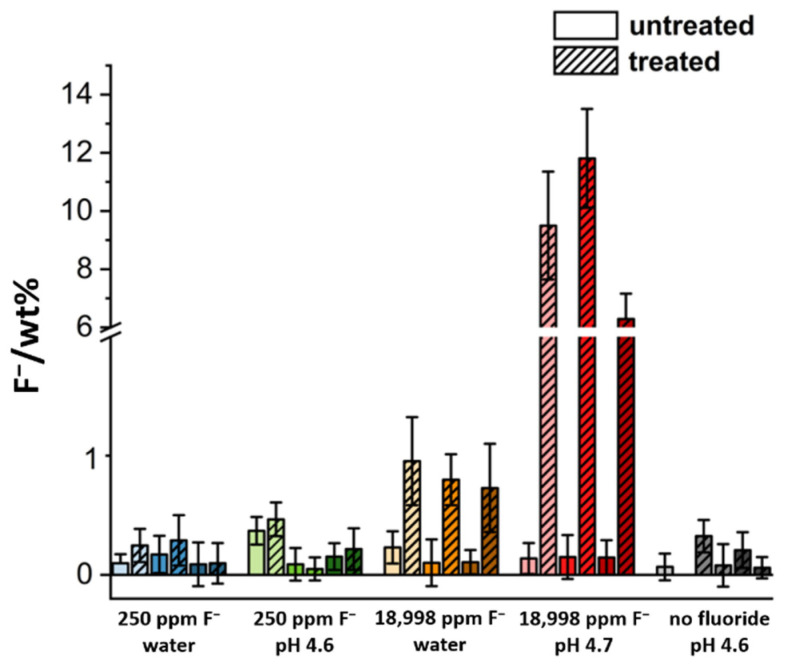
Average fluoride content of the different treatment groups as determined by EDX. For each group, three teeth were used. Shown are the fluoride contents in each individual tooth before and after immersion. In general, there was no significant change in the fluoride concentration after treatments with 250 ppm fluoride. At the very high concentration of 18,998 ppm fluoride (1 mol L^−1^), tooth enamel showed a highly significant fluoride uptake after the immersion, especially under acidic conditions.

After immersion, all teeth showed an increase of the fluoride concentration, but this was not always statistically significant. All results are given in Figure 2 and Table 1. A treatment with 250 ppm fluoride did not result in a significant change in fluoride content, neither at neutral nor at acidic pH. In contrast, teeth exposed to a fluoride content of 18,998 ppm showed a strong increase in fluoride content. Notably, the increase in fluoride concentration was about a factor of 10 higher at low pH.

In contrast, the immersion of teeth in fluoride-free citrate buffer (pH 4.6) did not show a significant decrease in the (low) fluoride content. An immersion of a tooth in water also showed no effect, i.e., the enamel surface was stable both in water (as expected) and in citrate buffer.

**Figure 3:**
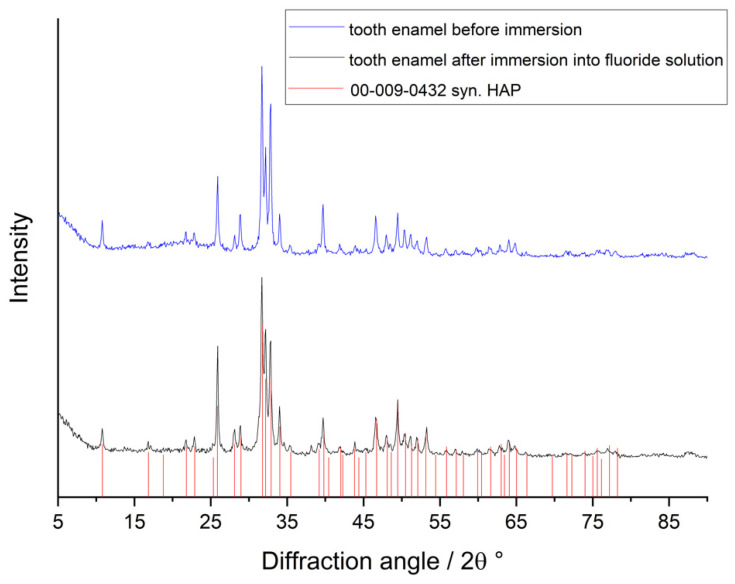
Representative X-ray powder diffractogram of tooth enamel before (**top**) and after immersion into 18,998 ppm fluoride solution in citrate buffer (pH 4.7; **bottom**). The red bars correspond to synthetic hydroxyapatite (PDF No. 00-009-0432).

The nature of the incorporated fluoride was assessed by X-ray powder diffraction. In principle, fluoride could be incorporated as ionic substitution into fluoroapatite [37] or precipitate in separate crystals as calcium fluoride [26]. Clearly, this effect will be strongest for samples with a very high fluoride uptake. Figure 3 shows X-ray powder diffractograms of untreated tooth enamel and of a sample that was treated with 18,998 ppm fluoride in citrate buffer. Under the latter conditions, 9 to 10 wt% of fluoride was found, i.e., well above the stoichiometric concentration of fluoride in fluoroapatite (3.77 wt%). There was no significant difference between the enamel phase before and after immersion, indicating an incorporation of fluoride into the hydroxyapatite tooth mineral as substituting ions (fluorohydroxyapatite) [37]. Note that hydroxyapatite and fluorapatite are almost indistinguishable by X-ray powder diffraction [38]. However, no other phase was detected, particularly no calcium fluoride (48.7 wt% fluoride) as reported after treatment of teeth with acidic fluoride gels [26].

**Figure 4:**
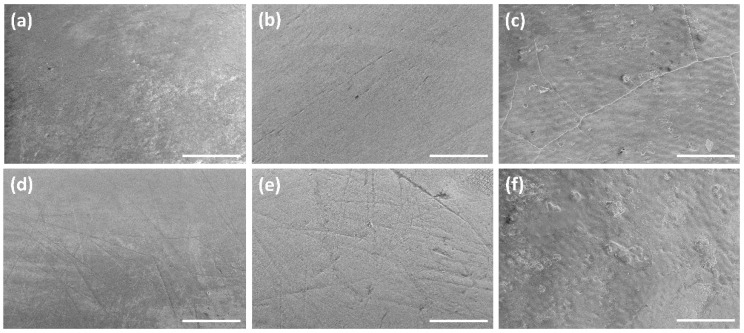
Representative SEM images of tooth enamel surfaces at a magnification of 2000× (**a**) without treatment (as-received), (**b**) after immersion in 250 ppm fluoride in water (pH 5.85), (**c**) after immersion in 250 ppm fluoride in citrate buffer (pH 4.6), (**d**) after immersion in 18,998 ppm fluoride in water (pH 7.76), (**e**) after immersion in 18,998 ppm fluoride in citrate buffer (pH 4.7), and (**f**) after immersion in in fluoride-free citrate buffer (pH 4.6). There was no significant change in the surface topography after immersion if the high variation between the teeth was taken into account. Scale bars 50 µm.

The tooth surface was also analyzed by scanning electron microscopy to detect morphological changes (Figure 4). We found a wide variability in the enamel surface topography as expected for the different provenience of the teeth. Cracks as well as scratches and irregularities were detected, due to previous tooth brushing, etc. Consequently, we compared the surface topography of each individual tooth before and after immersion. In no cases was a significant change in the topography of original and immersed teeth found.

## 4. Discussion

The treatment with a solution of 250 ppm fluoride for one minute did not significantly change the fluoride content of the tooth enamel, neither at neutral nor at acidic pH. This is in line with earlier reports where teeth were treated with fluoride-containing toothpastes [36,39,40]. The uptake of fluoride from toothpastes into a thin surface layer was reported to occur within a few minutes [41]. The fact that the immersion in acidic fluoride-free medium and also in water did not significantly change the fluoride content underscores the generally low fluoride concentration in teeth as reported earlier (see Ref. [14] for a recent overview of the literature).

In contrast, the treatment with an 18,998 ppm fluoride solution led to a significant uptake of fluoride especially at low pH, similar to studies where fluoride gels were applied [42], also at low pH [26]. This effect may be due to surface etching of the tooth enamel and partial dissolution/reprecipitation of fluoride-containing apatite. Even for these samples with about 10 wt% fluoride in the surface layer probed by EDX, globular CaF_2_ was never observed, neither morphologically by scanning electron microscopy nor crystallographically by X-ray powder diffraction. In particular, no other phase than apatite was found by diffraction, in line with earlier studies by Lelli et al. [39]. Apparently, the formation of such globules requires a longer immersion time or a more deeply etched tooth surface [35,43,44]. Thus, the chemical and crystallographic nature of the deposited fluoride-rich species remains unknown.

The comparatively low degree of fluoride uptake corroborates earlier results where teeth were immersed in fluoride-containing solutions (see Ref. [14] for a recent review, also on hydroxyapatite model surfaces). A fluoride uptake was observed by Scholz et al. after treatment of teeth with high-fluoride gels [26]. Lee et al. found fluoride in human teeth after treatment with fluoride strips [40]. Hjortsjö et al. also observed that the fluoride uptake in teeth was higher if the incubation occurred at low pH (down to pH = 1.6 to 3.1) [35].

Notably, the very thin fluoride-rich layer may just vanish in the much higher volume of abraded enamel used for X-ray diffraction. It must be emphasized that the surface layers of the teeth analyzed by EDX and by XRD are not identical. EDX is strongly surface-sensitive (probing the surface to about 100–200 nm depth) whereas XRD analyzes an abraded surface layer of a couple of 100 µm in thickness. Consequently, chemical and crystallographic nature of the fluoride-rich surface layers may well be different because the analyzed samples were basically not identical. Earlier studies by X-ray photoelectron spectroscopy have shown that the penetration depth of incorporated fluoride is only a few tens of nanometers [32,37,41,45], well in line with our results. Consequently, the fluoride-rich surface layer comprises only about 0.1 to 0.2 vol% of the abraded sample used for X-ray diffraction. This is well below the detection limit for crystalline phases studied by laboratory X-ray powder diffractometers, especially if poorly crystalline or nanocrystalline phases are present [46]. In addition, amorphous phases are not detectable by X-ray diffraction at all. Thus, we cannot rule out the presence of calcium fluoride (CaF_2_), but we also have no indication for its presence, except for the high fluoride content in the surface layer (9 to 10 wt%) which is beyond that possible for fluoroapatite (3.77 wt%). It should also be mentioned that the evidence reported in the literature for the formation of CaF_2_ was always based on elemental analysis by different methods [26,32,37,47] but never (so far) on a crystallographic analysis.

Fluoride-free prophylactic systems have been investigated in the last decade. It has been shown that they can be as efficient as fluoride-containing systems to prevent dental caries [8,48,49,50,51,52,53,54,55,56,57]. Thus, they may present a viable alternative to fluoride-containing agents in oral care.

## 5. Conclusions

Fluoride is present in human teeth only to a very small extent, mostly on the tooth surface. An immersion in fluoride-containing solutions led to an uptake of fluoride only if very high fluoride concentrations were applied (18,998 ppm fluoride, equivalent to 1 mol L^−1^), but no fluoride uptake was observed at a fluoride concentration of 250 ppm. Thus, very high fluoride concentrations in the surrounding medium (outside the range of most oral care products) are necessary to significantly increase the surface content of fluoride. The chemical and crystallographic nature of the fluoride-rich surface layer remains an open question due to its inherent thinness.

## Figures and Tables

**Table 1:** Average fluoride concentration and average changes in the fluoride concentration after the immersion in fluoride solutions.

Immersion Medium	Average Fluoride Concentration after Immersion/wt%	Average Change in Fluoride Concentration Compared to the Same Tooth before Immersion/wt%
250 ppm fluoride (water, pH 5.85, *N* = 3)	0.21 ± 0.10	+0.09 ± 0.07 (not significant)
250 ppm fluoride (citrate buffer, pH 4.6, *N* = 3)	0.24 ± 0.21	+0.04 ± 0.07 (not significant)
18,998 ppm fluoride (water, pH 7.76, *N* = 3)	0.83 ± 0.12	+0.68 ± 0.05 (significant, *p* < 0.0001)
18,998 ppm fluoride (citrate buffer, pH 4.7, *N* = 3)	9.2 ± 2.77	+9.06 ± 2.77 (significant, *p* < 0.0001)
Fluoride-free citrate buffer (pH 4.6, *N* = 3)	0.05 ± 0.04	−0.08 ± 0.15 (not significant)

## Data Availability

The data presented in this study are available on request from the corresponding author.
